# Head-Ache; Suppuration about the Left Ear; Disease of the Temporal Bone; Abscess in the Brain; and Remarks

**Published:** 1851-07

**Authors:** E. L. Ormerod

**Affiliations:** Cambridge.


### ARTICLE XIX.

*Head-ache; Suppuration about the Left Ear; Disease of the Temporal Bone; Abscess in the Brain; and remarks.* By E. L. Ormerod, M. B., Cambridge.

Case 30.—John B——, aged twenty-eight; admitted into St. Bartholomew's Hospital, Feb. 1846, under Dr. Hue. A carpenter, of a slight frame of body, temperate, always delicate, but not so as to call himself ill, till within the last three months, was admitted into the hospital a week before his death. His illness commenced with head-ache; then an abscess formed and burst behind his left ear; matter used also to be discharged from the meatus auditorius externus, with temporary relief to his deafness. The pain in his head was very severe, generally occurring in paroxysms; but it could not be distinctly made out that these paroxysms had any relation in point of time with the suppression of the discharge from the ear. He was so deaf that communication with him was almost limited to signs. He had cough, with abundant expectoration of a white froth, mixed with strings of puriform mucus, tenacious, and with the sickly smell so often noticed in the expectoration of phthisical patients, and orthopnœa; but his appetite, with all his sufferings, was good, almost to voracity, to the last.

*Body examined, eighteen hours after death.*—Limbs nearly all relaxed; blood generally coagulated. The skull was remarkably thin, the capacity of the right half greater than that of the left. The pericranium was extensively separated from the left temporal bone. The dura mater was generally thin and transparent, marked with a black spot in a point corresponding to the semicircular canals on the left side, where also a red granular growth had sprouted up and become attached to the brain and its membranes. Beneath, the bone was blackened, and a communication was discovered with the cavity of the internal ear, which contained a thick whitish pus. The coats of the left lateral sinus were thickened, and had a coagulum adherent to the lining membrane, and much thick pus lay beneath the dura mater, in the part where the sinus curves forwards on the petrous bone, the membrane being here separated from the bone for a considerable space. The under surface of the left middle lobe was of a sooty color, and fluctuating to the feel. On section, about an ounce of green stringy fetid pus was discharged from a cavity, apparently not at all implicating the corpus striatum or optic thalamus, surrounded by dark gelatinous walls. No pus exuded from the point of the brain above noticed as having adhered to the dura mater when the brain was removed from the skull. The rest of the brain was healthy, excepting that the convolutions of the vertex on the left side were flattened.

On examining the petrous bone the meatus internus appeared quite healthy, but the parts about the semicircular canals formed a cavity, divided into cells filled with pus, and covered with a dark gelatinous matter, which was, indeed, all that separated this cavity from the dura mater at the blackened point above alluded to. The cavity communicated by one opening with the external meatus, outside the tympanum, this membrane being entire and having the malleus adherent to it in a sound state; another led to the cellular tissue behind the external ear; and a third communicated freely with the collection of pus beneath the dura mater on the inside of the skull.

The heart was small, with an old tuft of fibrin at the apex, but chiefly remarkable for the entire absence of fat from the organ. There was an old pleural adhesion about the base of the right lung; in the left interlobular fissure lay a gelatinous looking mass, and behind, quite at the base, was about fifteen ounces of green fetid pus lying in the angle of junction of the ribs and diaphragm, inclosed in a flattened cyst of about 3.5 inches in breadth behind, but gradually tapering off towards the front. The walls internally were coated with thick pus and fibrin, and there was every appearance of the collection having existed there for a long period. Within, the lungs were emphysematous, the bronchi injected and loaded with pus and mucus, and the lower lobes more or less consolidated by pneumonia, the appearances being in some degree modified by the presence of tough cellular bands in the right base, and gelatinous-looking infiltration in the left.

The coats of the stomach and small intestines appeared remarkably thin; the lining membrane of the former was rather thickly coated with mucus. The mesenteric glands were generally large, hard and yellow; the mesentery and omentum containing no fat.

Other organs presented nothing particularly worthy of notice.

The first point that requires to be noticed in this case, is to regret that only so much of the lateral sinus as adhered to the portion of bone removed from the skull was examined; a further examination of this sinus, judging from what was seen, would have probably shown more extended traces of inflammation.

Considering the disease of the bone as the cause of the abscess in the brain, it was curious to observe how completely the cyst shut off the pus from any communication with the diseased bone. The same result of disease of the bone and dura mater, in producing abscess, entirely inclosed in the substance of the brain, is more strikingly shown, as in a more acute case in Dr. Abercrombie's third case of this nature, ("Diseases of the Brain," p. 35,) but yet more remarkably in Mr. Ormerod's "Clinical Collections," (p. 72,) where abscess of the brain followed upon mechanical injury of the bones of the head. The adhesion of the vascular growth from the dura mater to the brain is, perhaps, to be looked upon as the first step towards a spontaneous evacuation of the contents of the abscess, interrupted by death.—*London Lancet.*

